# Real-time speech MRI datasets with corresponding articulator ground-truth segmentations

**DOI:** 10.1038/s41597-023-02766-z

**Published:** 2023-12-02

**Authors:** Matthieu Ruthven, Agnieszka M. Peplinski, David M. Adams, Andrew P. King, Marc Eric Miquel

**Affiliations:** 1 Clinical Physics, Barts Health NHS Trust, West Smithfield, London, EC1A 7BE UK; 2 School of Biomedical Engineering & Imaging Sciences, King’s College London, King’s Health Partners, St Thomas’ Hospital, London, SE1 7EH UK; 3 Digital Environment Research Institute (DERI), Empire House, 67-75 New Road, Queen Mary University of London, London, E1 1HH UK; 4 Advanced Cardiovascular Imaging, Barts NIHR BRC, Queen Mary University of London, London, EC1M 6BQ UK

**Keywords:** Magnetic resonance imaging, Medical research

## Abstract

The use of real-time magnetic resonance imaging (rt-MRI) of speech is increasing in clinical practice and speech science research. Analysis of such images often requires segmentation of articulators and the vocal tract, and the community is turning to deep-learning-based methods to perform this segmentation. While there are publicly available rt-MRI datasets of speech, these do not include ground-truth (GT) segmentations, a key requirement for the development of deep-learning-based segmentation methods. To begin to address this barrier, this work presents rt-MRI speech datasets of five healthy adult volunteers with corresponding GT segmentations and velopharyngeal closure patterns. The images were acquired using standard clinical MRI scanners, coils and sequences to facilitate acquisition of similar images in other centres. The datasets include manually created GT segmentations of six anatomical features including the tongue, soft palate and vocal tract. In addition, this work makes code and instructions to implement a current state-of-the-art deep-learning-based method to segment rt-MRI speech datasets publicly available, thus providing the community and others with a starting point for developing such methods.

## Background & Summary

Use of real-time magnetic resonance imaging (rt-MRI) to visualise articulators and the vocal tract during speech is increasing in both research and clinical settings^1–11^. This increase is a result of the development of real-time MRI techniques with relatively high spatio-temporal resolutions and the unique ability of MRI to non-invasively acquire images of any view without using ionising radiation.

Typically, in real-time speech MRI, series of two-dimensional (2D) images of a midsagittal slice of the vocal tract are acquired^1–11^. State-of-the-art real-time speech MRI techniques can acquire such images at spatial resolutions of 2.4 × 2.4 mm^2^ or higher at temporal resolutions of 0.02 s or higher^12–14^. However, these techniques require highly specialised equipment and software, namely custom receive coils^13,14^ and/or specialised pulse sequences and image reconstruction methods^12–14^ that are not widely available, particularly in clinical settings. Real-time speech MRI techniques that only require more widely available standard equipment and software have been developed^15–18^. While these techniques image at lower spatio-temporal resolutions than state-of-the-art ones, the resolutions are nevertheless sufficient to capture the global motion of the main articulators during speech^2^.

To widen access to real-time speech MRI data and therefore stimulate research in the field, several speech MRI datasets that include series of 2D real-time images of a midsagittal slice of the vocal tract have been made publicly available^3,5–11^. Most of these datasets include image series of English^5–7^ or French^8,9^ speakers performing phonologically comprehensive speech tasks (i.e. speech tasks designed to include most phonemes in a wide range of contexts). The other datasets include image series of English speakers producing emotional speech^10^, repeating several speech tasks consisting of vowel-consonant-vowel sequences^11^, and imitating unfamiliar speech sounds^3^.

There is increasing interest in extracting quantitative information from 2D midsagittal MR images of the vocal tract^18–33^. In particular, there is interest in measuring the size, shape and motion of the vocal tract^18,19,22–32^ and articulators such as the soft palate^20,33–37^. To avoid the burden of manual measurements, methods to (semi-)automatically measure the size and shape of the vocal tract have been developed^38–46^ and methods to automatically measure the size, shape and motion of the soft palate are beginning to be developed^33,47–50^. Consistent with trends in other image analysis fields, most of the recently developed methods utilise convolutional neural networks (CNNs) and are therefore deep learning based^42–50^.

Deep-learning-based methods are achieving state-of-the-art performance in a wide range of image analysis fields including medical image analysis^51–53^. However, a requirement for the development of such methods is ground-truth (GT) segmentations as well as images. These GT segmentations are manually created, a process that is time-consuming and, particularly for biomedical images, requires input by specialists. While GT segmentations for 2D midsagittal MR images of the vocal tract have been created^46–50^, none are currently publicly available. The public availability of image sets with corresponding GT segmentations has been found to stimulate the development of state-of-the-art image analysis methods^54–56^.

This work makes two main contributions to the literature. First, by making real-time speech MRI datasets with corresponding GT segmentations publicly available, it begins to address a major barrier to the development of deep-learning-based speech MR image analysis methods. Second, by making code and instructions to implement a current state-of-the-art deep-learning-based speech MR image analysis method^47^ publicly available, this work provides the speech MRI community and others with a starting point for the development of such methods. Although, the MRI data made available has been previously used in published work;^47,50^ neither data nor segmentations had been published. Since the previous work was published, the GT segmentations have been revised. In particular, the boundary of the soft and hard palate is defined using a radiological interpretation (in line with the anterior wall of the sphenoid sinus) as opposed to a tissue basis as the soft and hard palate overlap. The manuscript also provides image acquisition and segmentation details should the reader wish to increase the size of the dataset. The main intention of these contributions is to facilitate and stimulate the development of novel state-of-the-art speech MR image analysis methods.

## Methods

### Subjects

Following approval by the Health Research Authority (HRA) and with support from the Joint Research Management Office (JRMO), five healthy adult volunteers (two females, three males; age range 24–28 years) participated in the study after providing informed consent to publish the data, in accordance with ethics committee requirements (LREC 22/PR/0058). The volunteers were fluent English speakers and had no history of speech and language disorders. The provided data is fully anonymised with no personal information remaining.

### Image acquisition

**Fig. 1:**
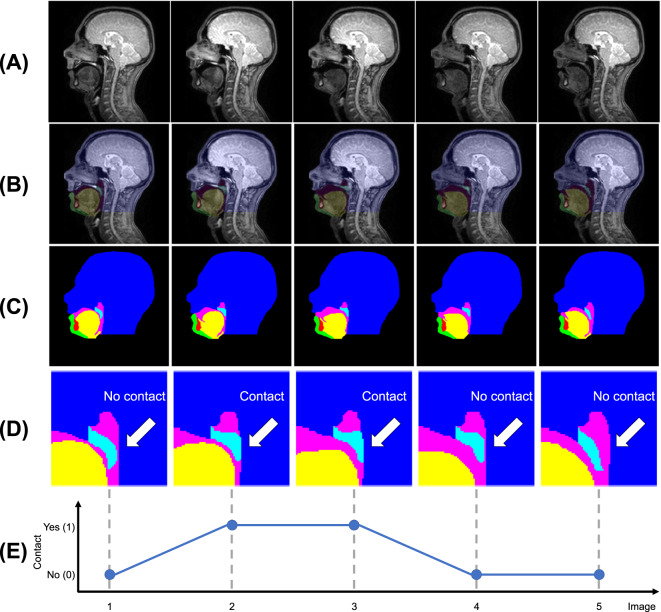
Five consecutive images from one of the magnetic resonance image series (**A**), corresponding ground-truth (GT) segmentations overlaid on the images (**B**), GT segmentations only (**C**), GT segmentations cropped around the soft palate with labels indicating if there is contact between the soft palate and posterior pharyngeal wall (**D**), and a line chart indicating if there is contact (Yes) or not (No) between the soft palate and posterior pharyngeal wall in each image in the series (**E**). The GT segmentations are of the head (dark blue), soft palate (light blue), jaw (green), tongue (yellow), vocal tract (pink) and tooth space (red) classes.

Each volunteer was imaged in a supine position using a 3.0 T TX Achieva MRI scanner and a 16-channel neurovascular coil (both Philips Healthcare, Best, Netherlands, software release 3.2) while they performed the following speech task a single time: counting from 1 to 10 in English. Images of a 10 mm thick mid-sagittal slice of the head were acquired using a steady state free procession (SSFP) pulse sequence based on the sequence identified by Scott *et al*.^15^ as being optimal for vocal tract image quality. Example images are shown in Fig. 1A. Imaging parameters are listed in Table 1. The acquired matrix size and in-plane pixel size were 120 × 93 and 2.5 × 2.45 mm^2^ respectively. However, k-space data were zero padded to a matrix size of 256 × 256 by the scanner before being reconstructed, resulting in a reconstructed in-plane pixel size of 1.17 × 1.17 mm^2^. To maximise the signal-to-noise ratio in the images, partial Fourier was not used. One image series was acquired per volunteer at a temporal resolution of 0.1 s. The volunteers were instructed to perform the speech task at a rate which they considered to be normal. Some performed the task faster than others and consequently not all series had the same number of images. The series had 105, 71, 71, 78 and 67 images each (392 images in total). Each series required a total scan time of 10.5, 7.1, 7.1, 7.8 and 6.7 s respectively. The process to identify the midsagittal plane was as follows. First, a localiser scan was performed that acquired series of 2D images of three perpendicular (approximately the axial, coronal and sagittal planes). Second, the images of the approximate sagittal planes were visually inspected and the plane of the image that most closely resembled a midsagittal plane was selected. Third, this plane was manually adjusted so that it passed through the nasal septum and between the two hemispheres of the brain in the images of the approximate axial planes.

**Table 1:** Imaging parameters of the Steady State Free Precession pulse sequence used to acquire the magnetic resonance image series, based on the pulse sequence identified by Scott *et al*.^15^ as being optimal for vocal tract image quality.

Parameter	Value
TR (ms)	2.0
TE (ms)	0.9
Flip angle (°)	15
Acquired/reconstructed matrix size	120 × 93/256 × 256
Acquired/reconstructed in-plane pixel size (mm^2^)	2.5 × 2.45/1.17 × 1.17
Slice thickness (mm)	10
Field of view (mm^2^)	300 × 230
Philips sequence name	FFE
SENSE factor	2
NSA	1
Actual WFS (pixel)/BW (Hz)	0.134/3240.4

Abbreviations are: repetition time, TR; echo time, TE; Fast Field Echo, FFE; sensitivity encoding, SENSE; number of signal averages, NSA; water fat shift, WFS; and bandwidth, BW.

### Velopharyngeal closure identification

**Fig. 2:**
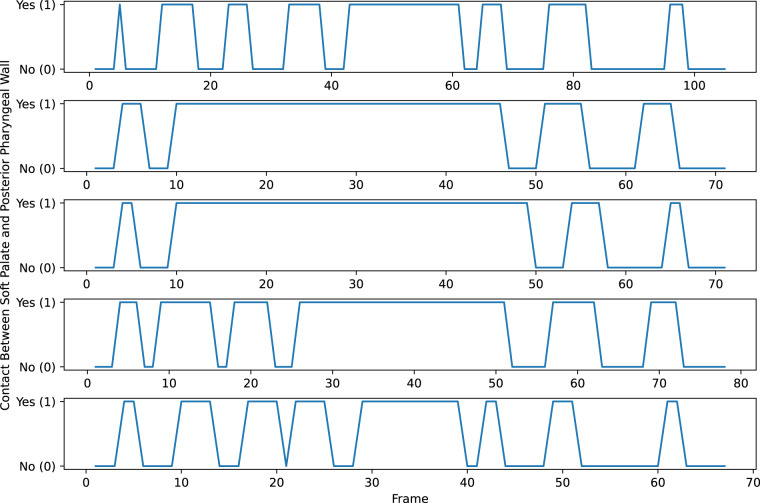
Ground-truth labels of the five image series. Each line chart represents a different series and has a different x-axis. Each peak in a line chart indicates a velopharyngeal closure.

Each image was visually inspected and labelled as either showing contact between the soft palate and posterior pharyngeal wall or not showing contact. Example images showing contact and no contact are shown in Fig. 1A. Line charts of the labels of each image series were created (example labels are shown in Fig. 1D) and visually inspected to determine the number of velopharyngeal closures shown in each series. In these charts, each peak represents a velopharyngeal closure, as consecutive images where the soft palate is in contact with the posterior pharyngeal wall show a single velopharyngeal closure. It can be challenging to determine if a 2D real-time MR image shows contact between the soft palate and posterior pharyngeal wall, especially if the soft palate is close to the posterior pharyngeal wall or if there is fluid surrounding the tissues, which can conceal tissue boundaries. To reduce the subjectivity of the labels, each image was independently labelled by four MRI Physicists. Raters one to four respectively had four, ten, two and one years of speech MRI experience. All the images were labelled again one month later by rater one. Intra- and inter-rater agreement was assessed by comparing the labels and the number of velopharyngeal closures determined from these labels. In cases where one rater disagreed with the others, the majority label was considered to be the GT label. In cases where only two raters agreed, raters one and two (those with the most speech MRI experience) jointly inspected the images and then reached a consensus on the labels for these images, similarly to how speech and language therapists jointly inspect videofluoroscopy speech image series in clinical practice in the United Kingdom. The consensus labels were used as the GT labels and the GT number of velopharyngeal closures was determined from these. Line charts of the GT labels are shown in Fig. 2.

### Ground-truth segmentation creation

**Fig. 3:**
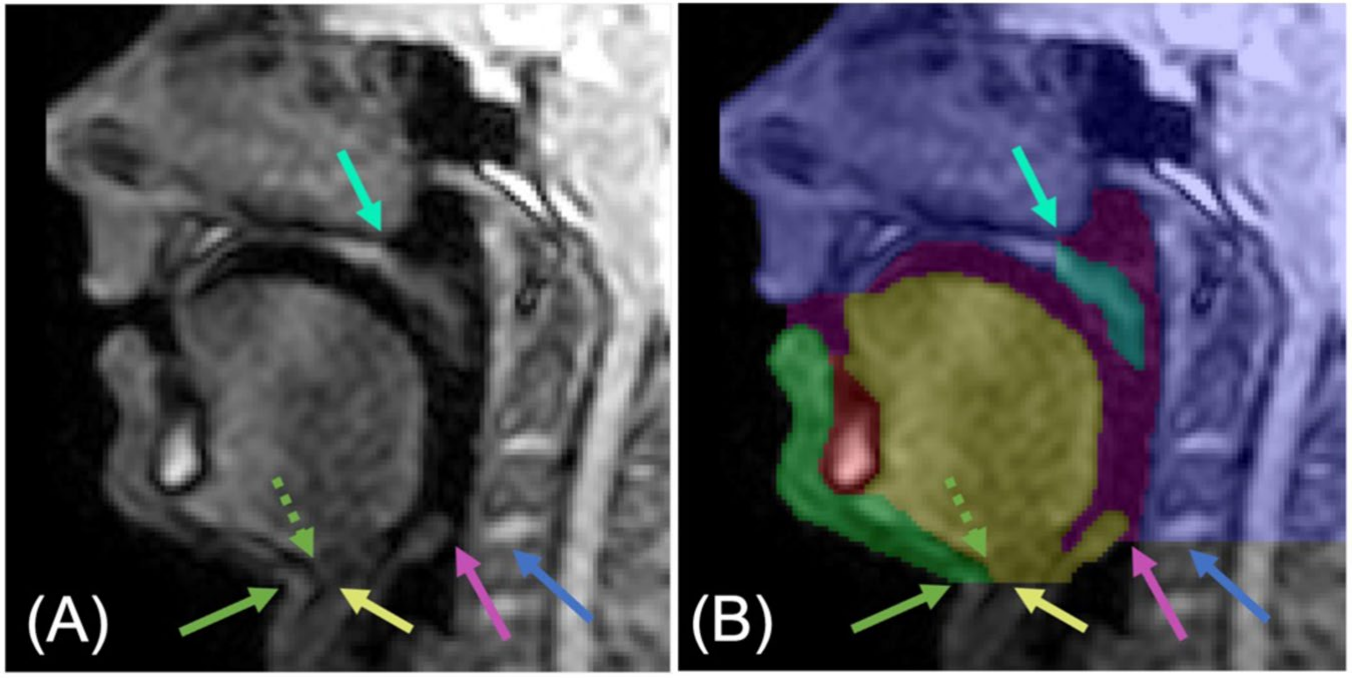
An image cropped to only show the vocal tract (**A**) with ground-truth segmentations overlaid (**B**). The dark blue arrows point to the inferior surface of the intervertebral disc between cervical vertebrae C3 and C4. The light blue arrows point to anterior wall of the sphenoid sinus. The dotted green arrows point to the anterior edge of the hyoid bone, while the solid green arrows point to where the neck meets the jaw. The yellow arrows point to the inferior boundary of the tongue class in the neck, while the pink arrows point to the inferior boundary of the vocal tract class.

GT segmentations were created by manually labelling pixels in each of the images. The segmentations consisted of six classes, each made up of one or more anatomical features. There was no overlap between classes: a pixel could not belong to more than one class. For conciseness, the classes were named as follows: head, soft palate, jaw, tongue, vocal tract and tooth space. However, the names of the head, jaw and tongue classes are simplifications. The head class consisted of all anatomical features superior to or posterior to the vocal tract. It therefore included the upper lip, hard palate, brain, skull, posterior pharyngeal wall, and neck. The jaw class consisted of the lower lips, the soft tissue anterior to and inferior to the mandible and the soft tissue inferior to the tongue. The tongue class included the epiglottis and the hyoid bone. Pixels not labelled as belonging to one of the classes were considered to belong to the background. Example GT segmentations are shown in Figs. 2 and 3B. The reasons for including the classes in the GT segmentations are given in Table 2.

**Table 2:** Reasons for including each class in the ground-truth segmentations of the magnetic resonance images of the vocal tract during speech.

Class	Reason(s) for inclusion
Head	*Primary:* segmentation of the posterior pharyngeal wall would enable automatic measurement of the distance between the soft palate and the posterior pharyngeal wall
	*Secondary:* segmentation of the upper lip would enable automatic lip motion tracking
Soft palate	Segmentation would enable soft palate shape and motion analysis, and also automatic measurement of the distance between the soft palate and the posterior pharyngeal wall
Jaw	Segmentation of the lower lip would enable automatic lip motion tracking
Tongue	Segmentation would enable tongue shape and motion analysis
Vocal tract	Segmentation would enable vocal tract shape analysis
Tooth space	Included so that there were no holes in the ground-truth segmentations, thus facilitating the post-processing of estimated segmentations

Wherever possible, the boundaries of the classes were chosen to be clear anatomical boundaries in order to minimise the subjectivity of the GT segmentations. Apart from the tooth space class, the majority of the class boundaries were easily distinguishable air-tissue boundaries. However, there were no clear anatomical boundaries for some sections of the class boundaries. Instead, the following artificial boundaries were devised for these sections. The two main goals when devising these boundaries were firstly to include only relevant anatomical features and secondly to minimise the subjectivity of the boundaries.

The inferior boundary of the head class in the neck was defined as the horizontal line parallel to the inferior surface of the intervertebral disc between cervical vertebrae C3 and C4 (see dark blue arrows in Fig. 3). This choice was made to exclude the inferior section of the neck in the head class as this section was not required for the desired analyses and would have otherwise increased the imbalance between the number of pixels in the head class and the other classes.

The boundary where the soft palate connects to the head class was defined to be in line with the anterior wall of the sphenoid sinus, and the boundary edge is perpendicular to the dark line that follows the edge of the hard palate (see light blue arrows in Fig. 3). The posterior boundary of the jaw class was defined as the anterior edge of the hyoid bone (see dotted green arrows in Fig. 3), while the inferior boundary of the jaw class in the neck was defined as the horizontal line intersecting the point where the jaw meets the neck (see solid green arrows in Fig. 3). The inferior boundary of the vocal tract class was defined in the same way as that of the head class (see pink arrows in Fig. 3), and the inferior boundary of the tongue class in the neck was defined in the same way as that of the jaw class in the neck (see yellow arrows in Fig. 3).

GT segmentations were created by the MRI Physicist with four years of speech MRI experience, using bespoke software developed in house and implemented in MATLAB R2019b (MathWorks, Natick, MA). GT segmentations were consistent with the GT label for the images: segmentations of the soft palate and posterior pharyngeal wall (part of the head class) were in contact for images labelled as showing contact and not in contact otherwise.

## Data Records

**Fig. 4:**
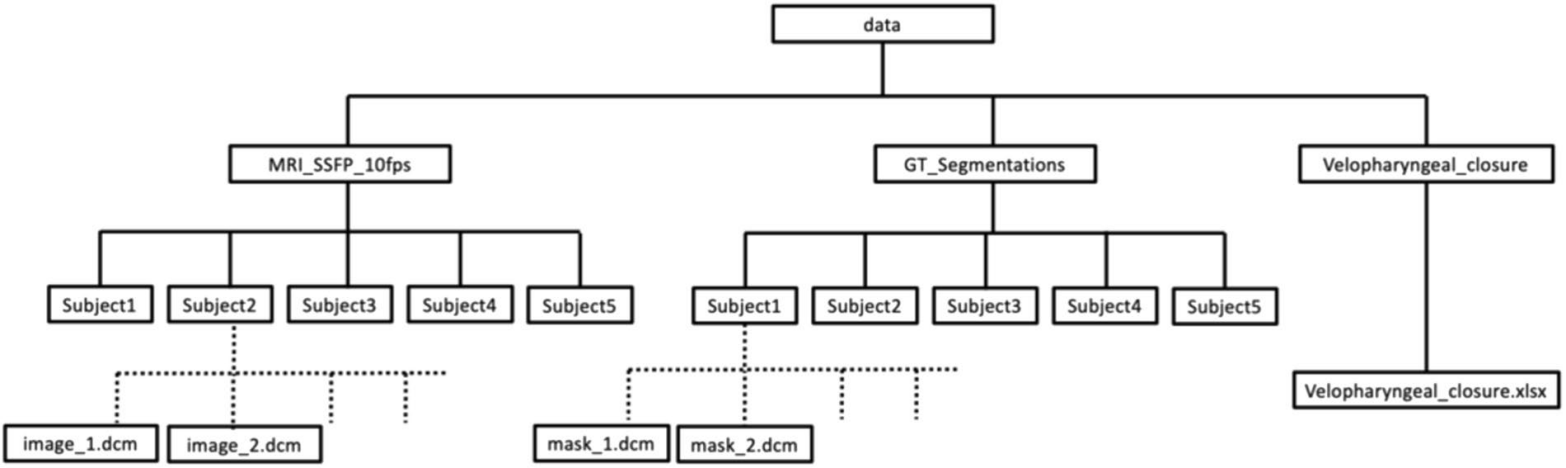
The structure of the directory containing the datasets on Zenodo.

The datasets are available on Zenodo^57^ (version 2) and consist of the five 2D real-time MR image series, GT segmentations and GT contact labels described in this article. The directory containing the datasets is structured in the way shown in Fig. 4. Images are contained in the *MRI_SSFP_10fps* folder. Within this folder, each subfolder contains the images of a different volunteer. Each image is saved as a separate anonymised DICOM file with name *image_N.dcm*. GT contact labels are saved in *velopharyngeal_closure.xslx*. The labels of each volunteer are saved in different sheets. The spreadsheet row corresponds to the image number (i.e. the label in row 1 is the label for image 1). A label of 1 indicates contact while 0 indicates no contact. GT segmentations are contained in the *GT_Segmentations* folder. Within this folder, each subfolder contains the GT segmentations of a different volunteer. Each GT segmentation is saved as a separate MAT file with name *mask_N.mat*. In each MAT file, pixels with the following values correspond to the following class:

- 0 = background
- 1 = head
- 2 = soft palate
- 3 = jaw
- 4 = tongue
- 5 = vocal tract
- 6 = tooth space

## Technical Validation

### Imaging

To increase the likelihood of acquiring images with a good image quality, a sequence based on the one identified by Scott *et al*.^15^ as being optimal in terms of image quality for 2D real-time vocal tract imaging at 3.0 T was used in this work. Nevertheless, before they were manually segmented, all the images were visually inspected by the MRI Physicists with four and ten years of speech MRI experience, to verify that air-tissue boundaries between the vocal tract and articulators were clearly visible and that no artefacts obscured the articulators in the images.

### Velopharyngeal closure analysis

**Fig. 5:**
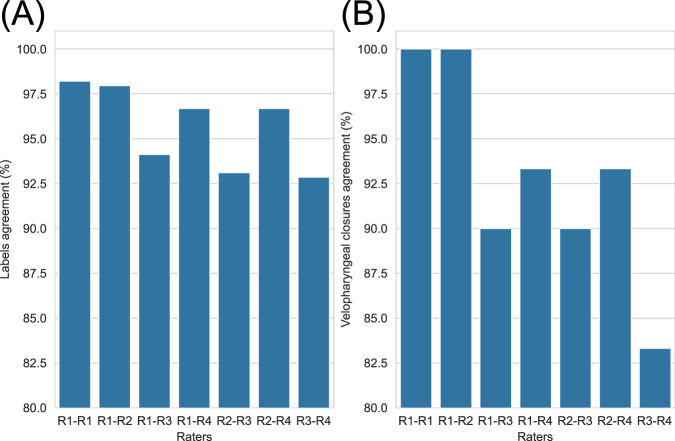
Intra- and inter-rater agreement in the labels of the 392 images (**A**) and in the velopharyngeal closures (**B**).

The subjectivity of the GT closure labels was investigated by assessing the intra- and inter-rater agreement in the labels. As shown in Fig. 5, there was intra-rater agreement in the labels for 98.2% (385 of 392) images and in all 30 velopharyngeal closures. In three image series, intra-rater agreement in the labels was 100% (220 of 220) images, while in the other two image series intra-rater agreement in the labels was 97.0% (65 of 67) and 95.2% (100 of 105) images respectively. All label differences were for images at the start or end of a velopharyngeal closure, where the soft palate is close to or in contact with the posterior pharyngeal wall. Such discrepancies affected the durations of velopharyngeal closures but not the number of velopharyngeal closures.

**Fig. 6:**
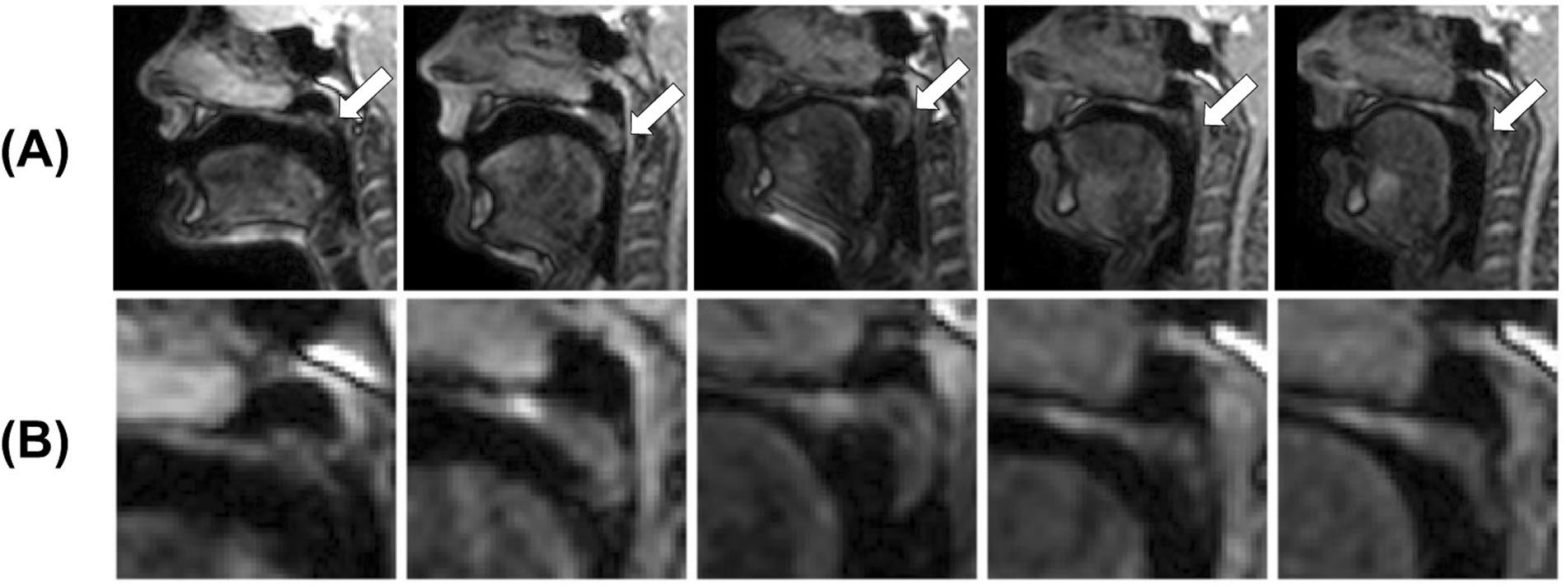
Images cropped to only show the vocal tract (**A**) and soft palate (**B**) where only two out of four raters agreed on the label.

There was complete inter-rater agreement in the labels of 357 of 392 (91.1%) images and in 25 of 30 (83.3%) velopharyngeal closures. All label differences were for images where the soft palate was close to or in contact with the posterior pharyngeal wall. In two image series, there was complete inter-rater agreement in all 12 velopharyngeal closures. In the other three image series, there was complete inter-rater agreement in 5 of 6 (83.3%), 3 of 4 (75.0%) and 5 of 8 (62.5%) velopharyngeal closures respectively. As shown in Fig. 5, raters one and two had the highest inter-rater agreement, with agreement in the labels of 384 of 392 (98.0%) images and in all 30 velopharyngeal closures. There was inter-rater agreement between at least three raters in the labels of 385 of 392 (98.2%) images and in all 30 velopharyngeal closures. Figure 6 shows images where inter-rater agreement in labels was low. In all five cases where there was inter-rater disagreement in a velopharyngeal closure, one of the raters considered there to be two closures instead of one.

### Ground-truth segmentation creation

The largest to smallest GT segmentation classes in terms of number of pixels are the head with a median of 23633 pixels per segmentation (ranging between 21093–21353 pixels), tongue with 2075 pixels (1936–2298), vocal tract with 1153 pixels (754–1560), lower jaw with 945 (827–1133), soft palate with 237 pixels (187–268) and tooth space with 160 pixels (104–187).

**Fig. 7:**
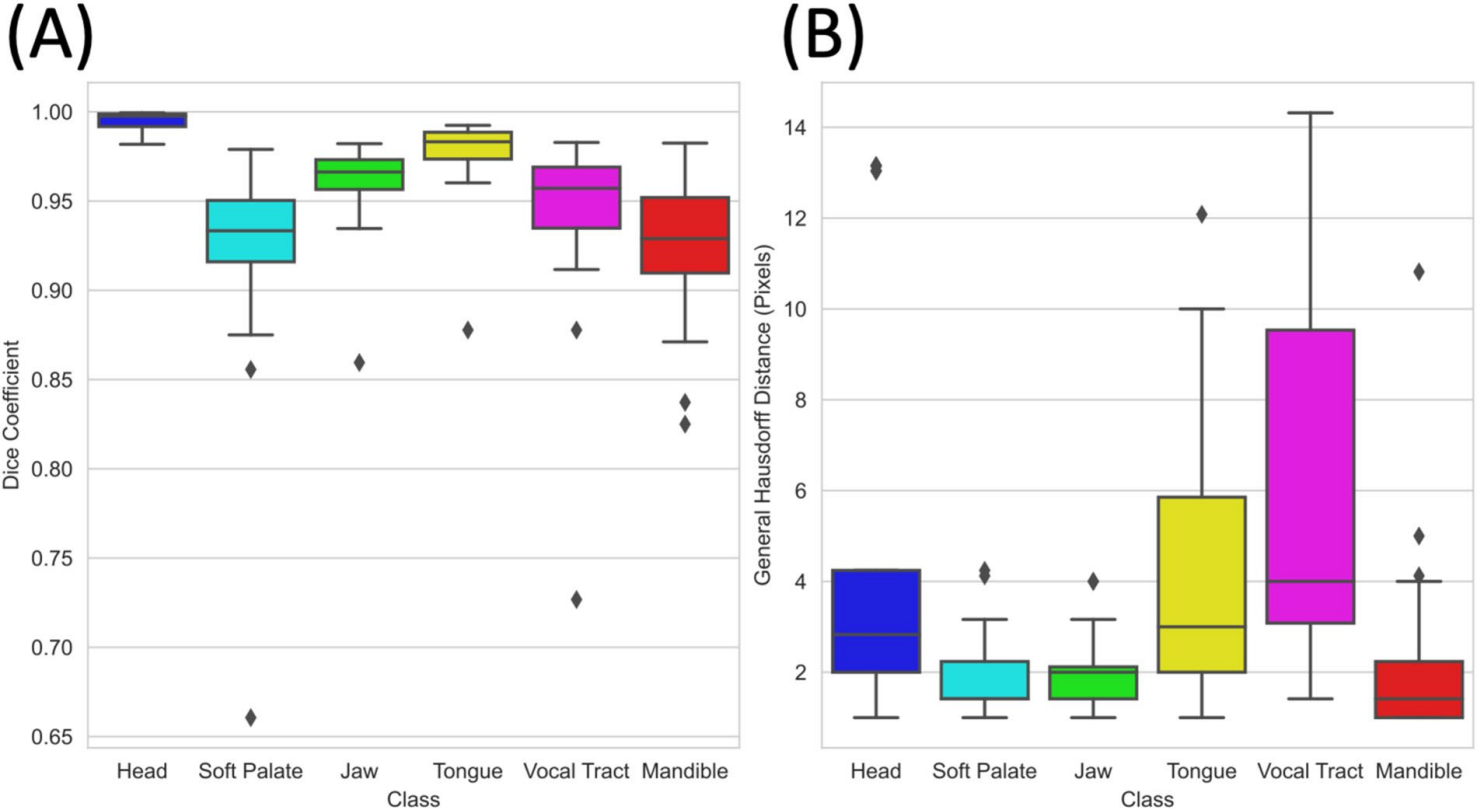
Intra-rater agreement in the ground-truth segmentations, quantified using the Dice coefficient (**A**) and general Hausdorff Distance (**B**).

To enable investigation of intra-rater agreement and therefore uncertainty in the segmentations, the Physicist created GT segmentations again for seven (approximately 10%) randomly chosen images in each series. The agreement was quantified using two widely-used metrics in the medical image analysis community^58^: the Dice coefficient (DSC) and the general Hausdorff distance (HD). The median intra-rater agreement was 0.965 and 2.24 pixels respectively. As shown in Fig. 7, intra-rater agreement was highest for segmentations of the head class with a median DSC of 0.997 and a median HD of 2.8 pixels, while intra-rater agreement was lowest for segmentations of the tooth space and soft palate classes, with median DSC of 0.929 and 0.933, and a median HD of 1.41 and 1.41 pixels, respectively. Segmentations of the soft palate class had the largest range in DSCs, closely followed by segmentations of the tooth space.

## Usage Notes

The datasets described in this article can be used to develop methods to segment speech MR images. Code to train and evaluate such a method^47^ using the datasets is publicly available in the following GitHub repository: https://github.com/BartsMRIPhysics/Speech_MRI_2D_UNet (software licence: Apache-2.0). The datasets and the code provide the speech MRI community and others with a starting point for developing such methods. In conjunction with other datasets, the datasets described in this article could be used to develop methods to segment a broader range of speech images acquired using different imaging techniques. The datasets could also easily be modified to enable the development of methods to analyse air-tissue boundaries in speech images, such as methods^38–45^.

This dataset contains images of only five healthy volunteers all acquired using the same MRI scanner and sequence combination. Although the low number of subjects is a limitation, it was previously shown to be sufficient to develop and train segmentation networks^47^ or to inform registration^50^. Clear instructions for acquisition and segmentations are given for readers who wish to increase the size of the dataset.

## Data Availability

The code that accompanies this article is publicly available in the following GitHub repository: https://github.com/BartsMRIPhysics/Speech_MRI_2D_UNet (software licence: Apache version 2.0). The repository contains already trained versions of a state-of-the-art speech MR image segmentation method^47^ that are ready to use immediately. These versions were trained using the datasets described in this article. The repository also contains instructions and Python code to train and evaluate new versions of the method using the datasets described in this article. The code is designed to allow users to choose several important training parameters such as the training and validation dataset split, the number of epochs of training, the learning rate and the mini-batch size. In addition, the code is designed to be compatible with any dataset as long as it is organised and named in a specific way. The repository contains Python code to check that the datasets are not corrupted and are organised and named in the specific way required by the segmentation method, as well as Python code to perform the image pre-processing required by the method, namely normalising the images and saving the normalised images as MAT files.
